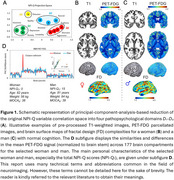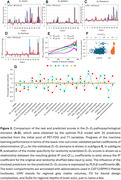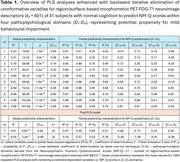# Predictive Modelling of Transformed NPI‐Q Scores Using Region‐ and Surface‐Based Morphometry of MRI‐PET‐FDG Images in Healthy Controls

**DOI:** 10.1002/alz70856_103555

**Published:** 2025-12-26

**Authors:** Rafael Dolezal, Zdenek Linha, Matej Seifert, Tejasvi Ravi, Pavla Brennan Kearns

**Affiliations:** ^1^ 2nd Medical School, Charles University, Prague, Czech Republic, Czech Republic; ^2^ National Institute of Mental Health ‐ CZ, Klecany, Czech Republic

## Abstract

**Background:**

Despite advances in machine learning, neuroscientists continue to face challenges related to the informational non‐univocity of morpho‐functional indices for detecting the early phases of neurodegeneration. Mild Behavioural Impairment (MBI), conceptualized as a set of specific pathopsychological phenomena, is investigated herein to shed light on the potential implications of metabolic and structural brain changes in cognitively healthy individuals before irreversible neuronal degeneration leads to significant consequences for their lives.

**Method:**

Utilizing the resources of the ADNI database, 41 randomly selected cognitively normal subjects with T1 scans, PET scans for 2‐deoxy‐2‐[^18^F]fluoroglucose (FDG) and reduced (*D_1_‐D_4_*) questionary‐based neuropsychiatric scores (NPI‐Q) were included into this preliminary study on the predictability of MBI signs, employing region‐based and surface‐based brain‐morphometry techniques (Figure 1). After processing the PET/T1 neuroimages, which involved PVC‐PET, DARTEL‐warping and Desikan‐Killiany‐based parcellation with internally normalized FDG‐SUVRs, 597 functional and geometrical brain descriptors were generated. Along with common nuisance parameters and white matter T1‐hypointensities, the dataset (41x601) was explored with developed algorithms for partial‐least‐square regression (PLS), enhanced with backward iterative elimination of uninformative variables (BIEUV). Predictivity and specificity of the PLS model was confirmed especially with the cross‐validated *Q^2^_LOO_* coefficients and by testing the model performance deterioration on scrambled *D_1_‐D_4_* scores (Figure 2).

**Result:**

After 576 iterations, which removed 97% of variables, 25 stable predictors survived and provided a statistically significant model for simultaneous prediction of new NPI‐Q‐related scores representing four pathopsychological domains (i.e. unrest, agitation, disbalance, psychosis) (global *R^2^* = 0.91, *p* = 4.0e^‐15^, global leave‐one‐out cross‐validated *Q^2^_LOO_* = 0.56) (Table 1). The best and worst predictabilities were found within the agitation (*D_2_*) and psychosis (*D_4_*) domains, respectively. Importantly, the detected variance of PLS‐*β*‐coefficients suggests that many of the monitored brain parts may induce opposite responses in different pathopsychological domains (e.g. FDG uptake in hippocampus). Nevertheless, the synergic effect of all 25 selected predictors must be considered simultaneously to retain their predictivity.

**Conclusion:**

A computational diagnostic tool for predicting NPI‐Q‐related scores in four pathopsychological domains using neuroimaging data from cognitively normal subjects was presented in this study. The pathopsychological scores were reproduced by a PLS model through the BIEUV‐based reduction of the PET‐FDG and T1 data.